# Barrier removal and dynamics of intermittent stream habitat regulate persistence and structure of fish community

**DOI:** 10.1038/s41598-022-05636-7

**Published:** 2022-01-27

**Authors:** Krzysztof Kukuła, Aneta Bylak

**Affiliations:** Department of Ecology and Environmental Protection, University of Rzeszow, ul. Zelwerowicza 4, 35-601 Rzeszów, Poland

**Keywords:** Environmental sciences, Limnology

## Abstract

Barrier effects observed in the presence of weirs are exacerbated by low water levels. We conducted a 10-year study to assess the ecological effects of stream restoration while analysing the possibility of a seasonal lack of hydrological continuity, with multiple measurements before and after restoring stream structural continuity. The research hypothesis assumes that in intermittent streams, there would be little or no change in the fish community downstream the barrier before *vs*. after barrier removal, and a significant change upstream the barrier before *vs*. after. Our results indicate, that by removing small barriers, their detrimental effects on the longitudinal passage of riverine fishes and fish assemblages can be rehabilitated. In the wet season, fish migrants from the mainstem river appeared in the downstream section of the stream. Stream intermittency, however, placed a habitat filter over the assemblage. Thus, after barrier removal, only two small-bodied fish species that tolerate periodic oxygen deficiencies and rising water temperatures gradually shifted upstream and formed stable populations. We emphasize, that we should not refrain from restoring the longitudinal continuity of intermittent streams, because they periodically provide fish valuable refugia and can also be a source of new generations and strengthen fish populations in mainstem river.

## Introduction

The proper functioning of flowing water ecosystems is conditioned by continuity in several dimensions^1^: longitudinal (i.e., upstream–downstream), lateral (i.e., floodplains–channel), and vertical (i.e., surface water–groundwater), as well as temporal; this is illustrated by Ward's concept of the four-dimensional nature of lotic ecosystems^2^. Increasing human interference and intensive exploitation of flowing waters has led to the transformation of the physical characteristics of channels and the degradation of flowing water ecosystems^3–6^. One of the most important problems is river regulation. Its consequence is increased riverbed erosion^7^, resulting in a decrease in groundwater level (vertical disturbance)^8^, and the disruption of longitudinal continuity of rivers^9,10^. Longitudinal and lateral barriers can cause substantial changes in the fish fauna^11,12^. In particular, this applies to long-distance migrating species, such as diadromous^13^ and potamodromous fish species^14,15^, as these barriers cut them off from their breeding sites^13^. In addition, in a situation of interrupted continuity, i.e., such as in case of barrier effect, individual fish may be isolated or the fish population may get fragmented^16,17^.

Barrier effects observed in the presence of weirs, culverts, and fords are often exacerbated by low water levels^11^, and because of climate warming, low flows are even more severe^18,19^. In certain areas, this causes streams to dry completely or partially, as has been observed in North America^20^, South America^21^, South Africa^22^, Asia^23^, and Australia^18^, and in Europe^24–27^. Consequently, perennially flowing streams are transformed into intermittent streams^26^. Such streams are dynamic ecosystems that transition between flowing and dry phases. During the wet season, if stream habitats are well-preserved, intermittent streams can be a spawning ground for fish^28,29^, and the presence of young-of-the-year fish (YOYs) is a quantifiable result of successful spawning, as well as a good indicator of ecological quality of streams^30^. Some fish from mainstem watercourses may also periodically use intermittent streams as a feeding ground^31,32^, or as a refuge from predation and deteriorating environmental conditions^33,34^. In the dry season, water in the intermittent stream may be present in the form of isolated pools, or dry completely^27^. Therefore, fish in intermittent streams are seasonally exposed to harsh and even extreme habitat conditions, including increased water temperatures and oxygen deficiencies in the residual water (i.e., the isolated pools)^35,36^. Even with only the partial drying of stream channels, if fish cannot reach these refugia owing to interruption of stream continuity, they remain in these ecological traps and die^35,37^.

After the stream is dry, fish may re-populate a rewetted reach if they survive in more water-rich isolated pools^32,37,38^. However, the most important natural mechanism for the restoration of fish fauna seems to be the recolonisation of streams from the mainstem rivers, and exchange of migrants between subpopulations^39,40^. The importance of maintaining longitudinal continuity throughout the dendritic fluvial system is currently increasingly emphasised^3^. Remedial measures have also been taken^15^. Demolition of dams, dismantling of weirs, and construction of fish passes are increasingly used in restoration practice^41^. Therefore, it is possible to restore the continuity of river systems in the longitudinal dimension^42^. After such treatments, fish may settle in streams that are ‘open to colonisation’^43^. Relatively less frequently, however, attempts are made to restore historical flows in watercourses which, as a result of anthropogenic activities, transformed from perennial to intermittent streams^7^. This aspect of watercourse restoration seems much more difficult to implement, and perhaps is even impossible^8^.

The experiment followed a before-after design, with multiple measurements before and after implementing activities aimed at restoring stream structural continuity with a known impact date (i.e., barrier removal). We investigated how the presence/absence of barriers interacts with seasonal flow conditions to affect fish distribution and abundance. To do this, we sampled the fish community twice in each of the ten years; during the late summer dry season and during the spring wet season. We hypothesised that in intermittent streams, there would be little or no change in the fish community downstream the barrier before *vs*. after barrier removal, and a significant change upstream the barrier before *vs*. after.

The research questions addressed were as follows: i) what was the effect of barrier removal on the longitudinal distribution of the riverine fish community?; ii) how did seasonal flow conditions interact with barrier removal to affect the dynamics of the longitudinal distributions of fishes?; iii) which factors determine the relative stability of fish communities in intermittent streams after barrier removal?; and iv) what are potential recommendations that can be made for environmental managers?.

## Results

### Fish sampling

**Figure 1:**
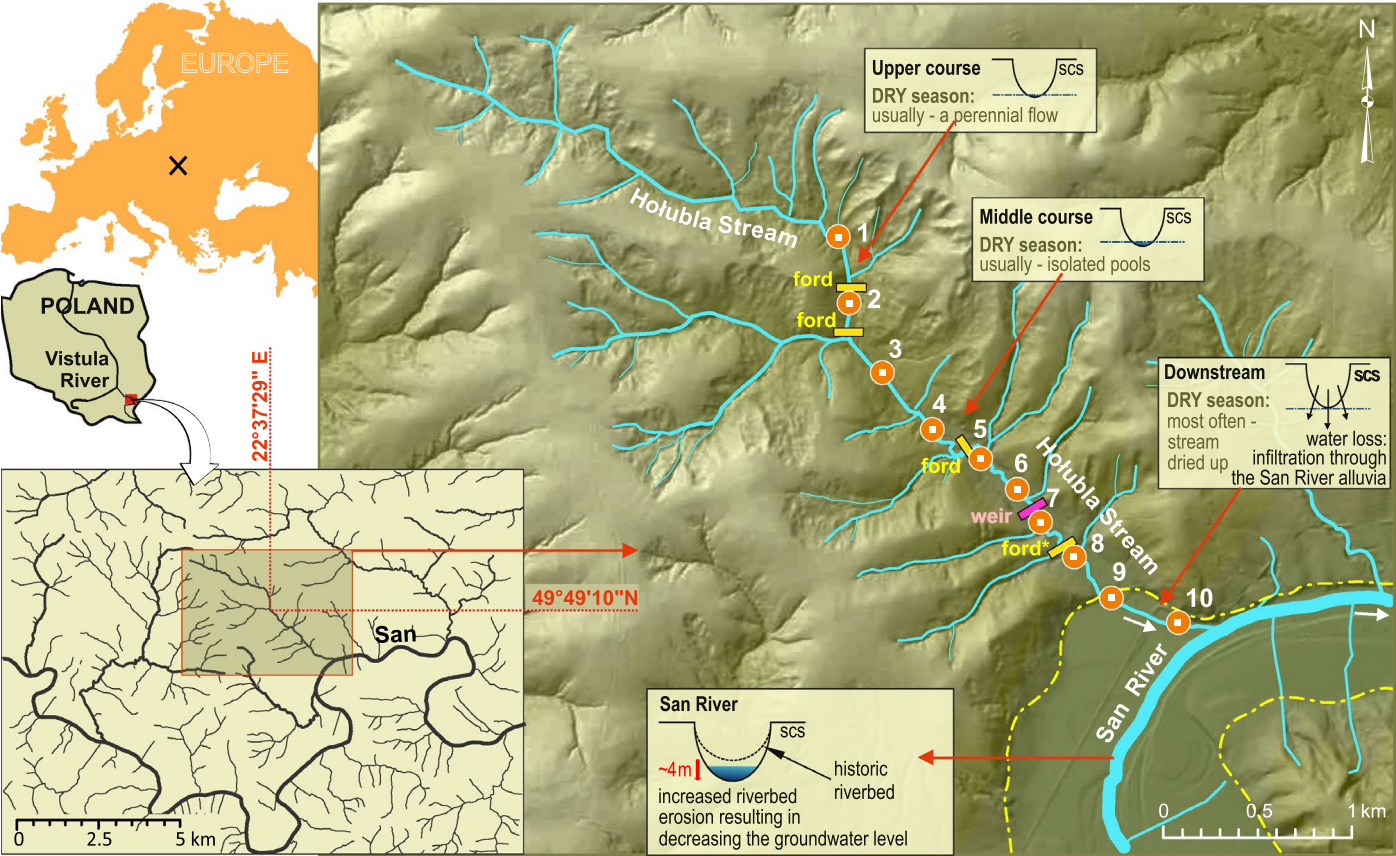
Map of the study area showing the sampling sites in the stream catchment; dash-dotted line—approximate boundaries of the San River alluvia; rectangles—transverse barriers, i.e., weir or fords; circles – sampling sites; scs – simplified cross-section, *—the most downstream barrier. In 2013, all the barriers were removed; UPSTREAM reach = the upstream reach of the most downstream barrier, i.e., upper and middle course of Hołubla Stream, sites 1–7; DOWNSTREAM reach = the downstream reach of the most downstream barrier, i.e., downstream course of Hołubla Stream, sites 8–10. CorelDRAW v. 21.0, ArcMap v.10.7.1) https://mapy.geoportal.gov.pl/wss/service/PZGIK/NMT/GRID1/WMTS/HypsometryAndShadedRelief.

A total of 4255 fish representing 10 species was caught at all sites in the Hołubla Stream (Fig. 1). The most numerous were the common minnow (*Phoxinus phoxinus*) and the stone loach (*Barbatula barbatula*), constituting approximately 75% of all fish caught. The chub (*Squalius cephalus*), gudgeon (*Gobio gobio*) and brown trout (*Salmo trutta fario*) accounted approx. 12%, 8% and 2%, respectively. The remaining species, i.e., dace (*Leuciscus leuciscus*), barbel (*Barbus barbus*), spirlin (*Alburnoides bipunctatus*), European bitterling (*Rhodeus amarus*), and perch (*Perca fluviatilis*), had a share of less than 1%. In the mainstem river (San River), a total of 31 fish species were found (Table 1). All species caught in the Hołubla Stream were also found in the San River.

**Table 1:** Fish species in the San River on the river section near the mouth of the Hołubla Stream (based on catches from 2012, 2017, 2019). The family names are in bold, ^A^ – alien species.

Scientific name	Common name
**Cyprinidae:**	
*Abramis brama*	Bream
*Alburnoides bipunctatus*	Spirlin
*Alburnus alburnus*	Common bleak
*Aspisus aspius*	Asp
*Barbus barbus*	Barbel
*Barbus carpathicus*	Spotted barbel
*Barbus waleckii*	Wałecki barbel
*Blicca bjoerkna*	White bream
*Carassius carassius*	Crucian carp
*Carassius gibelio* ^A^	Prussian carp^a^
*Chondrostoma nasus*	Nase
*Gobio gobio*	Gudgeon
*Leuciscus cephalus*	Chub
*Leuciscus leuciscus*	Common dace
*Phoxinus phoxinus*	Common minnow
*Pseudorasbora parva* ^A^	Stone moroko^a^
*Rhodeus sericeus*	European bitterling
*Romanogobio albipinnatus*	White-finned gudgeon
*Romanogobio kesslerii*	Kessler's gudgeon
*Rutilus rutilus*	Roach
*Vimba vimba*	Vimba bream
**Balitoridae:**	
*Barbatula barbatula*	Stone loach
**Cobitidae:**	
*Sabanejewia baltica*	Golden loach
**Salmonidae:**	
*Salmo trutta* m. *fario*	Brown trout
**Percidae:**	
*Gymnocephalus cernuus*	Ruffe
*Perca fluviatilis*	Perch
**Esocidae:**	
*Esox lucius*	Nothern spike
**Cottidae:**	
*Cottus gobio*	European bullhead
**Gadidae:**	
*Lota lota*	Burbot
**Siluridae:**	
*Silurus glanis*	European catfish
**Anguillidae:**	
*Anguila anguila*	European eel

### Impact of barrier removal on fish communities

**Figure 2:**
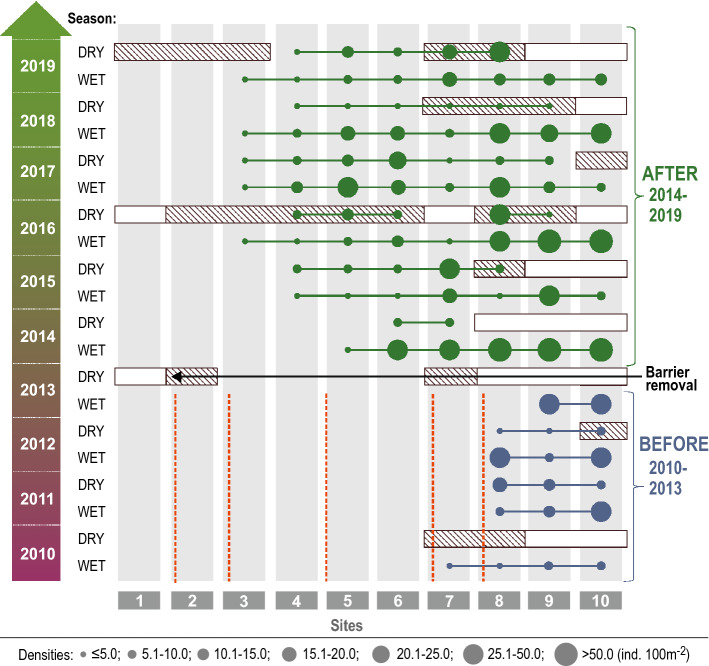
Ranges of fish sampled from the Hołubla Stream; white rectangle—dry stream channel; hatched rectangle—dry riffles and isolated pools in the stream channel; dashed line—transverse barriers; circles—generalised fish densities, a solid lines—fish ranges.

**Figure 3:**
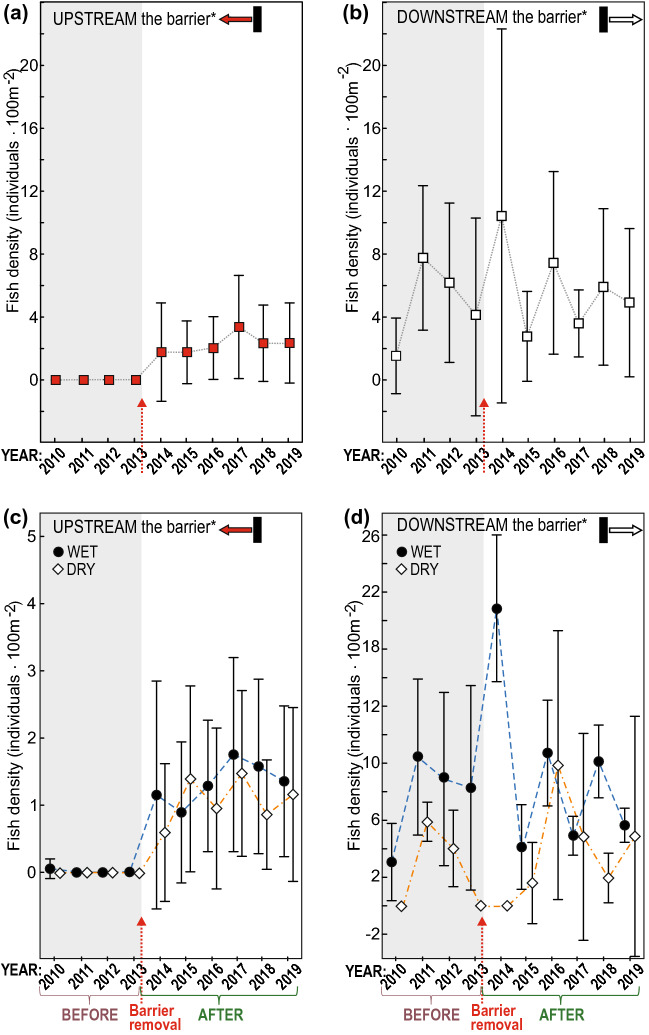
Time series for average total fish density; the symbols (circles and squares) indicate the mean value; the whiskers indicate the + /− standard deviation.

In the BEFORE period fish were detected only in the downstream course of the Hołubla Stream (Fig. 2), and the fish range ended at the most downstream barrier. After the removal of the barriers, the range of fish increased significantly. In the spring of 2014, the first year after ‘opening to colonisation’, the fish were already present at site 5. In the following years, the range of the fish increased by almost 1 km upstream, and fish were caught at sites 4 and 3 (Fig. 2). In the UPSTREAM reach (i.e., the upstream reach of the most downstream barrier; upper and middle course of Hołubla Stream; sites 1–7; see Fig. 1), the differences in fish density between BEFORE and AFTER periods were significant (U-test = -6.72, *p = *0.000001) (Fig. 3a), while in the DOWNSTREAM reach, the differences between BEFORE and AFTER in general fish density were not significant (U-test = -0.31, *p = *0.754) (Fig. 3b).

The results of PERMANOVA analysis of variations in fish assemblage structure in the sites throughout the research period revealed that the factors Reach, Season, Period, and Site, as well as almost all the interaction terms were significant (Table 2). The pairwise tests for the UPSTREAM reach indicated that fish communities at sites 4–7 did not differ significantly (Table A1a), and for the DOWNSTREAM reach no differences were recorded for sites 8–10 (Table A1b).

**Table 2:** PERMANOVA results based on Bray–Curtis distances of abundance data for assemblages of fish in two reaches (UPSTREAM the barrier and DOWNSTREAM the barrier), in the two sampling periods (BEFORE and AFTER barrier removal), and at two seasons (WET season and DRY season).

Source of variation	d.f	SS	pseudo-F	*P*
Reach	1	38,233	11.21	0.001
Season	1	19,412	18.61	0.001
Sampling period	1	16,084	4.16	0.028
Site (Reach)	8	3412	5.34	0.001
Site (Reach) x Season	8	8344	1.63	0.035
Site (Reach) x Sampling period	8	30,928	6.05	0.001
Reach x Season	1	15,156	14.53	0.001
Reach x Sampling period	1	9366	2.42	0.081
Pooled terms	170	108,550		
Total	199	283,930		

Pooled terms –Season x Sampling period; Reach x Season x Sampling period; Site(Reach) x Season x Sampling period; 999—permutations; SS—sum of squares.

### Interactions of presence/absence of barriers with seasonal flow conditions ***vs***. fish communities

In the BEFORE period when the stream was completely filled with water, the fish range ended at the most downstream barrier. In the DRY season, the part of the stream channel in the downstream course dried up, and the fish died or moved to the mainstem river. In the summer of 2013, the Hołubla Stream dried up and became completely fishless. After the removal of the barriers, in summer, the downstream course of the Hołubla Stream was drying up, but the fish were constantly present at sites located in the non-drying course of the stream, where the fish survived even in very dry summers, inhabiting non-drying pools (Fig. 2). At sites 3–7 after barrier removal (AFTER), the fish showed a slightly lower density in the DRY season than in the WET season. The average total fish density in the DRY season was 6.42 ind. 100 m^-2^, and 9.69 ind. 100 m^-2^ in the WET season, but the differences were non-significant (U-test = −1.16, *p = *0.245, Fig. 3c). In the DOWNSTREAM reach (site 8–10) in the WET and DRY seasons, the fish densities between BEFORE and AFTER periods were not significant (Fig. 3d). However, in the reach DOWNSTREAM reach, in the WET season, the fish density was significantly higher than in the DRY season (U-test = − 4.99, *p = *0.000001).

**Figure 4:**
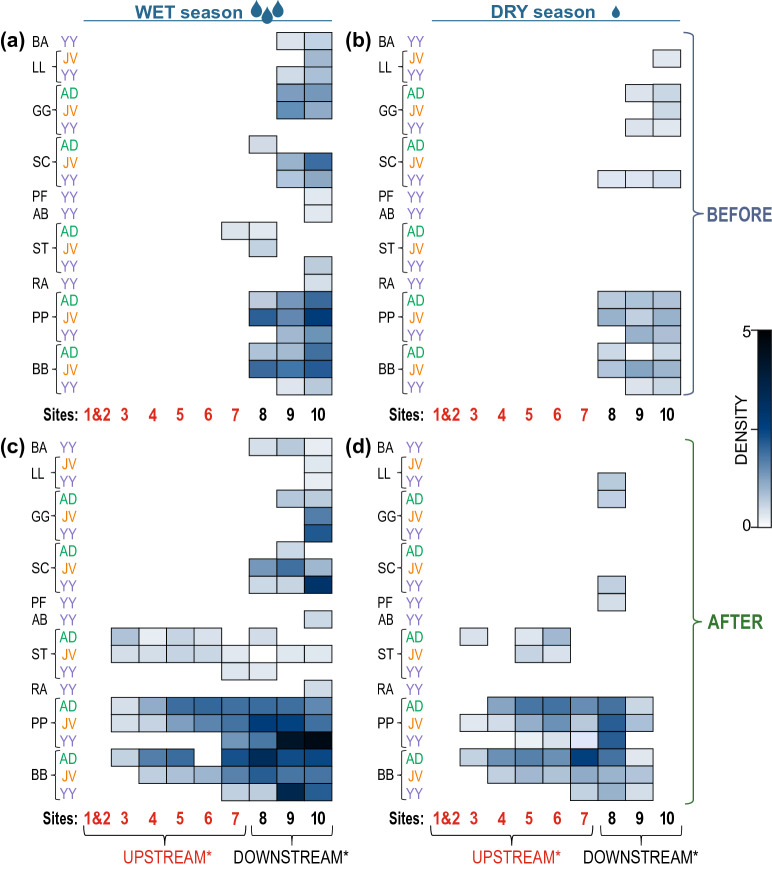
Shade plot of log(x + 1) transformed age class densities for all fish species at sites in the Hołubla Stream catchment; AB, *Alburnoides bipunctatus*; BA, *Barbus barbus*; BB, *Barbatula barbatula*; GG, *Gobio gobio;* LL, *Leuciscus leuciscus*; PF, *Perca fluviatilis;* PP, *Phoxinus phoxinus*; RA, *Rhodeus amarus*; SC, *Squalius cephalus;* ST, *Salmo trutta fario*; YY, young-of-the-year (fry during their first year of life); JV, juveniles; AD, adults; *—the most downstream barrier.

In the BEFORE period (2010–2013), no fish were found in the UPSTREAM reach of the most downstream barrier (Fig. 4a,b). The exception was site 7 in the spring of 2010; in the WET season, one relatively large (19.3 cm in total length) brown trout was caught. Ten fish species were caught at sites 9–10, and only four species were caught in the slightly upstream site 8. The most abundant fish in the WET season was the common minnow and stone loach, with an average density of 12.7 ind. 100 m^−2^ and 8.5 ind. 100 m^−2^, respectively, both at site 10. In the case of both species, all age classes, including YY individuals, were present at sites 9 and 10 (Fig. 4a). In spring, in the downstream section, YY and juvenile cyprinids (family Cyprinidae) were abundant: chubs, daces, and barbels. Gudgeons were represented by individuals older than YY. Brown trout appeared only in the WET season. In the DRY season, in the downstream section of the Hołubla Stream, the common minnow, gudgeon, and stone loach were represented by all age classes, but their mean densities were lower than the densities noted in the WET season (Fig. 4a,b).

No fish were found in sites 1 and 2 in the AFTER period (2014–2019). During the summer, sites 10 and 9 were dry or there were only isolated pools; hence, there were either no fish (site 10) or fish were in low densities (site 9). However, in the WET season (in spring) in the downstream section, there were many fish (Fig. 2). Ten species were identified. Young common minnows were very frequent. At site 10, this species had an average density of over 30 ind. 100 m^-2^, including 20 YY ind. 100 m^-2^. Only three species were found at site 7 upstream: common minnow, stone loach, and brown trout (Fig. 4b,d). At site 7, all age classes of the common minnow and stone loach were present. However, adult fish appeared mainly upstream. The most upstream site with fish was the site 3, where there were adult stone loach, adult and juvenile common minnows, and larger brown trout (from 14.8 to 26 cm), in small quantities (Fig. 4c,d).

At the sites in group GrU, the average Bray–Curtis similarity in the analysed group was low, reaching 20% (Table A2). The highest AvSim values (i.e. highest contribution to site similarity) were determined for adult minnows and stone loaches (cumulative contribution 77%). At sites in GrD1 (DRY season), the average Bray–Curtis similarity in the analysed group was very low, reaching 12%, and the highest AvSim values were recorded for adult and juvenile minnows and juvenile stone loaches. At sites in GrD2 (WET season), the average Bray–Curtis similarity in the analysed group was 38%, and the highest AvSim values were observed for adult and juvenile minnows and stone loaches (Table A2).

### Habitat factors determining fish communities after barrier removal

**Figure 5:**
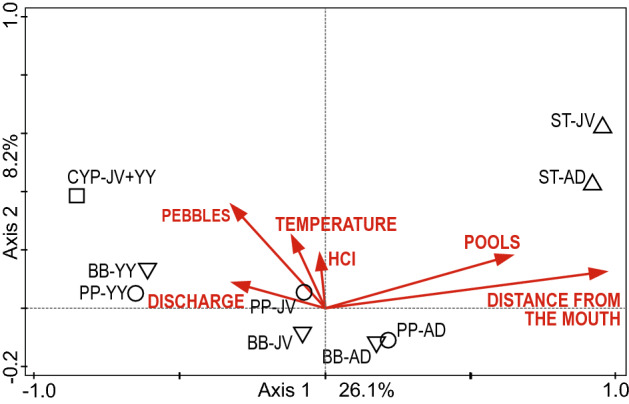
Canonical correspondence analysis biplot. BB, *Barbatula barbatula*; PP, *Phoxinus phoxinus*; ST, *Salmo trutta fario*; CYP – riverine cyprinids, belonging to the species associated with the mainstem river; YY, young-of-the-year (fry during their first year of life); JV, juveniles; AD, adults.

CCA analysis indicated the significance of all ordinance axes (pseudo-F = 5.2, *p = *0.001). Axis 1 (pseudo-F = 2.6, *p = *0.001) was highly correlated with the variables DISTANCE FROM THE MOUTH (DFM) and POOLS. Axis 2 was correlated the most with DISCHARGE. The CCA analysis showed that the DFM, POOLS, and DISCHARGE variables significantly explained the variability in fish abundance (Table 3). Adult and juvenile brown trout exhibited a high correlation with DFM and POOLS. The occurrence of YY in the two species, stone loach and common minnow, was strongly negatively correlated with DFM. Young individuals of riverine cyprinids (CYP-JV + YY) were strongly positively correlated with DISCHARGE and negatively correlated with DFM. The common minnow and stone loach from classes AD and JV, located in the centre of the ordination plot, did not show significant correlation with any of the variables (Fig. 5).

**Table 3:** Ranking of the environmental variables importance to the fish density AFTER barrier removal, based on their simple and conditional term effects on ecological factors.

Variables	Simple term effects			Conditional term effects		
	Explained %	pseudo-F	P	Explained %	pseudo-F	P
Distance from the mouth	21.3	17.8	0.001	21.3	17.8	0.001
Pools	10.1	7.4	0.001	3.4	2.9	0.009
Discharge	3.9	2.7	0.031	2.3	2.0	0.059
Pebbles	3.6	2.5	0.037	2.1	1.8	0.104
Temperature	1.9	1.3	0.223	3.5	3.2	0.003
HCI	1.1	0.7	0.596	2.7	2.6	0.017

## Discussion

**Figure 6:**
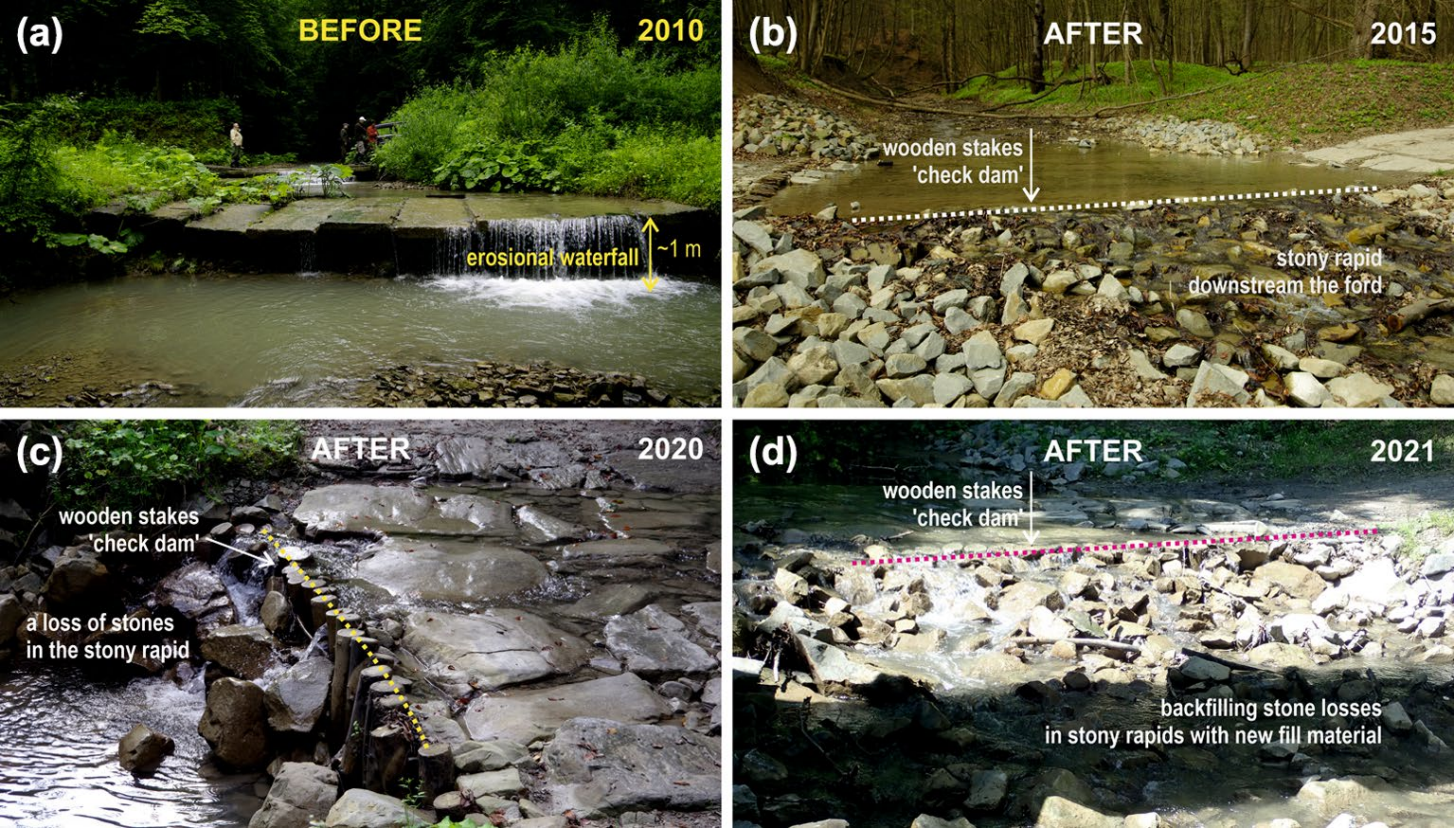
Road-stream crossing in the Hołubla Stream, in the stream reach between site 4 and 5; (**a**) before, (**b**) two years after, (**c**) seven years after channel restoration works, and (**d**) backfilling stone losses in stony rapids with new fill material (*photo K.Kukuła*).

Low barriers in the bed of the analysed small submontane stream can be considered the main cause of fish loss in the period before the removal of barriers (prior to 2013). Both stone weirs and fords made of concrete slabs with erosional waterfalls downstream were barriers to fish passage*.* During the 4 years of the pre-restoration period, in the stream section upstream the most downstream barrier, we caught only one large brown trout. It was probably one of the few fish that was able to overcome this barrier using the higher water level in the stream (Fig. 6a). Barriers, even low-heads, place constraints on the longitudinal passage of riverine fishes, leading to strong and significant effects on the assemblage composition of streams. They prevent fish from reaching the spawning grounds in the upper part of the streams and their tributaries^44–46^. These structures also block recolonisation, preventing the upstream movements of migrants from the lower section of the stream and the mainstem river^40^. Upstream compensation migration is also an important mechanism after floods when fish are carried downstream by floodwater, and they can move upstream again only in a free-from-barrier stream^14,47^.

However, these barriers can be removed, and their detrimental effects on riverbed geomorphology can be rehabilitated, such that fish assemblages can be rehabilitated. After removing the barriers, in the spring of 2014, there were numerous fish in the downstream reach of the Hołubla Stream, and we called this stage of recolonisation ‘the great fish run’. Several freshwater fish species make substantial movements into newly wetted reaches to rapidly recolonise all suitable habitats^32,48^. In the Hołubla Stream, migrants from the mainstem river appeared in the lower section of the stream including fry and juvenile chubs, daces, barbels, spirlin, brown trout, European bitterling, alongside juvenile and adult gudgeon, common minnows, and stone loach. This showed that native fishes quickly recolonise reaches previously cut off by barriers, and the presence of fry may be evidence of recruitment.

However, since the nineteenth century, the studied stream has been fundamentally modified because of the regulation of the mainstem river and climate warming. From historically cold water, the permanent stream (Archive of the Krasiczyn Forest District, personal communication P. Włodek) gradually turned into an intermittent stream, with shallow riffles and runs that warm up quickly in summer. Thus, although longitudinal continuity was restored in the stream by removing the barriers, stream intermittency placed a habitat filter over the assemblage. As described by the hypothesis of environmental filters, the composition of each assemblage resulted from species-specific niche differences in adaptation responses, and compositions evolved along environmental gradients^49,50^.

**Figure 7:**
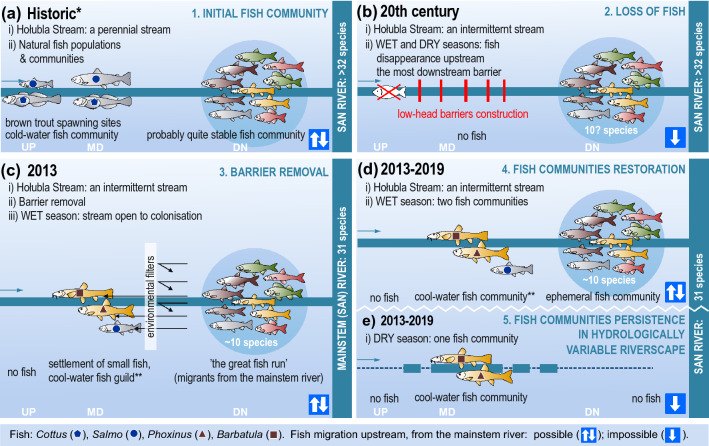
Conceptual model of (**a**,**b**) fish community responses to anthropogenic activities, and (**c**,**d**) the temporal and seasonal dynamic of fish community recovery following structural stream continuity restoration; UP—upstream course; MD—middle course, DN—downstream course of the stream; *- according to Dynowska and Maciejewski^67^, **- with very few brown trouts; fish guilds—according to literature^52,53,82^.

A functional discontinuity^51^ occurs seasonally and quite regularly (Fig. 2). This caused the lower stream section, most often only in spring, accessible to fish that formed only a seasonal community; such a community can be called ephemeral. Each year in spring, *'*de novo' fish formed only a temporary community comprising a set of the most abundant species from a pool of species from the mainstem river. According to the 'environmental filters' hypothesis^49,50^, the habitat requirements of the majority of species from this ephemeral fish community in the downstream section of the Hołubla Stream did not allow them to reach and permanently settle in the upstream section (Fig. 7). Only species with certain characteristics could recolonise the studied stream. The pool of fish species found in the mainstem river included two small-bodied species, feeding on benthic invertebrates and tolerating periodic oxygen deficiencies and rising water temperatures^52,53^. They were common minnow and stone loach, for which the habitats offered by the small intermittent tributary were appropriate^53,54^. Taking advantage of the hydrological continuity in spring, the common minnow and stone loach gradually shifted slightly upstream. Despite its small size, the common minnow is known for its relatively distant movements. It undertakes spawning migration in spring, during which it moves several kilometres upstream^55,56^. Even the species considered to be a short-distance migrators with low swimming capacities, the stone loach^52^, move several kilometres^45^. In the colonisation and recolonisation of stream habitats, environmental filters and competitive mechanisms play a major role in shaping communities^57,58^. In the mainstem river, the San River, the fish assemblage was rich in species because of a high habitat diversity that offered niches to a large number of species^59^.

Although the fish habitats in the mainstream San River are very diverse, habitats typical of common minnow and stone loach are scarce^53,59^. In mainstem rivers, the common minnow chooses slow-flowing habitats, near bank shallows and pools^45,52^. As there are many ‘beneficiaries’ of the limited area of such habitats, a lot of interspecific competition is generated^57,58^. In the San River, the shallow, near-riverbank habitat patches with many hiding places preferred by the minnow were also chosen by other cyprinids (Kukuła K. and Bylak A., unpubl. data). Conversely, the Hołubla Stream seasonally offers good habitat conditions without competitors and, additionally, without predators. Under historical conditions, adult common minnows in the Hołubla Stream were accompanied by the predatory brown trout. Presently, the brown trout is sparse in the middle course of the stream. In newly intermittent rivers, the loss of top fish predators, and subsequent modifications of species interactions are expected to have cascading effects on community structure, composition, and resilience^36,60^. Constantz^61^ argued that rewetted intermittent reaches generally offer substantial benefits through decreased predation and competition, and increased food availability.

It seems that in the surveyed intermittent stream, owing to habitat segregation, a fish community with weakened competition has developed. One of the species forming the community, the common minnow, represents the fish living mostly in the water column, and the second, stone loach, is typically bottom dwelling^62^. Data on the habitat and food preferences of both species^52,63^ demonstrate that they occupy slightly different niches. These species spawn in spring, mainly in May and June. The common minnow is a lithophilous fish, and the stone loach is a psammophilous fish^52^. The habitat conditions in the Hołubla Stream seem to be appropriate for spawning and egg development. The water is well oxygenated and there are patches of gravel and sand in the bottom substrate. The presence of YY fish, both minnows and loaches, in the Hołubla Stream suggests the recruitment of new generations in this stream. The spawning efficiency appears to be related to the duration of the flow conditions.

Additionally, drying in the middle course of the Hołubla Stream meant that the stream was divided into isolated pools. This created new problems. The fish remained in isolated pools and were cut off from the downstream and upstream reaches of the stream, becoming ensnared in ecological traps^36,37^. Isolated pools are places where fish densities initially increase, and therefore, intra- and inter-competitive influences intensify^36,64^. Moreover, the temperature may be periodically high, and dissolved oxygen low^36^, and fish trapped in shrinking stream pools may die^35^. The traps cause loss of populations, but the fish that survive can be the source of new generations, and additionally, populations can be augmented by downstream migrants^31,32^ in spring.

Our research was also aimed at formulating recommendations for water managers and decision-makers. It seems that the periodic lack of hydrological connectivity with the mainstem river can be minimised by increasing groundwater retention and slowing the flow in the river. Such restoration measures have not yet been introduced. When assessing the hydromorphological parameters and functional continuity of the study stream, we also focused on the processes related to the transformation of the riverbed sections rebuilt as a part of revitalisation works by the flowing water. In the Hołubla Stream, after the completion of the restoration works, especially in the dry season, in the rebuilt sections of the stream, the problem of water 'escape' in newly created stone rapids became noticeable. In this small stream, when the bed was only partially filled with water, the water disappeared under the stones and was not visible on the surface. Over time, however, we noticed positive changes with the gradual filling of the space between the stones with gravel and sand. Consequently, this enabled the formation of a stream of water visible on the surface of the rapids, and thus, they will become even more fish friendly.

Conversely, we observed that small erosive waterfalls began to form downstream of nature-friendly rapids (Fig. 6b,c). We strongly suggest that managers should monitor the stability of the rebuilt stream bed and control the tendency of rapids to form downstream waterfalls that could transform them into a new barrier to fish movement. It is strongly recommended to backfill the loss of stones in the stony rapids (i.e., voids, hollows) with new fill material (Fig. 6d) until the stability and natural dynamics of the stream bottom are restored, and balance is achieved between erosional-, transport- and depositional-dominated stream reaches. It is essential for maintaining stream connectivity and ensuring a stable passage for the fish^62,65^.

## Conclusions

Some negative changes to the environment caused by humans may be practically irreversible, such as the mainstem river channel cutting into the alluvial deposits and the consequent lowering of the groundwater level. Such changes cause the drying of the downstream sections of tributaries in dry seasons and the transformation of perennial streams into intermittent ones. Often, there are no fish in such streams, and some managers associate the lack of fish with the partial drying of the streams.

We identified three main stages of intermittent stream re-colonisation by fish, i.e., 1) ‘opening to colonisation’ and ‘the great fish run’, 2) settlement of non-drying reaches of the stream by small and cool-water fish species, and 3) seasonal formation of an ephemeral fish community in the downstream section of the stream and seasonal exchange of migrants between the stream and the mainstem river (Fig. 7). Thus, we demonstrated that if longitudinal continuity is maintained or restored in intermittent streams, the fish fauna that inhabit these areas may be relatively permanent. This is possible when the natural mosaic of habitats offering favourable feeding and spawning grounds is preserved, and the natural sequence of pools that provide water refuges for dry seasons is maintained. The hydrological connection of intermittent tributaries with the mainstem river, renewed during the wet season, ensures the exchange of fish migrants. Fish from a mainstem river can strengthen fish populations in the tributaries and vice versa^31,32^. Therefore, the main factors determining the relative stability of fish communities in intermittent streams can be considered longitudinal continuity in the entire river system and hydrological connectivity, at least seasonally, between tributaries and the mainstem river.

Finally, our results should help water managers and stakeholders in decision-making and identification of appropriate restoration practices, barrier removal, fish pass construction, and streambed-level restoration, even for small intermittent streams. The systematic restoration of the structural continuity of the entire fluvial system, along with small tributaries^42^, is a priority that is also included in the Water Framework Directive (WFD)^66^. In addition to restoring longitudinal continuity, it is also extremely important to maintain and restore the natural pool-riffle sequences, as in dry seasons, pools may be of key importance for the survival of stream fish. Although intermittent streams are typically poorly represented in biomonitoring programs that are implemented to characterise the ecological status according to the WFD^27^, we should not refrain from restoring their longitudinal continuity. Intermittent streams periodically provide fish refugia from predation and abundant feeding grounds. Additionally, by offering spawning grounds and habitats for fry growth, they can even be a source of new generations thereby strengthening fish populations in the mainstem river.

## Methods

### Ethics statement

A sampling permits were issued by the Marshal Office of the Podkarpackie Voivodeship following approval by the Regional Directorate for Environmental Protection. Research project was approved by the Department of Biology and Agriculture’s Committee for Research Ethics. The research was conducted under license to operate electroshocking tools and license to perform animal investigations according to legislation on the protection of animals and the recommendations of the International Council for Laboratory Animal Science (ICLAS).

### Study area

The Hołubla Stream catchment (5.89 km long, catchment area 8.68 km^2^) is located in the Dynowskie Foothills (Polish section of the Carpathians), and is a part of the Middle San River and the Upper Vistula Basin^67^ (Fig. 1, Table A3—detailed study area characteristics). Owing to the drainage effect of the San River and the lowering of the groundwater level, most of the small tributaries of the San River, including the Hołubla Stream, in their downstream sections near the mouth to the mainstem river, dried up under dry seasons (Fig. 1). According to historical data, until the 1930s, the Hołubla Stream was a perennial cold-water stream (Archive of the Krasiczyn Forest District, P. Włodek personal communication). The Hołubla Stream catchment area (Fig. 1) belongs to the Carpathian climatic region^68^ (Table A3b, A4), and has a high natural value (Table A3c). In the 1970s, one 3-m-high stone weir and four concrete slab fords were built across the Hołubla Stream streambed. With time, because of an increase in channel erosion downstream of the fords, waterfalls have been created (Fig. 6a). In 2013, these barriers were removed. Two concrete fords were rebuilt as natural stone fords (Fig. 6b), with wide crevices between stones, allowing the fish to move upstream. The other two fords were replaced by large metal arch culverts, which did not interfere with the natural base of the stream. Downstream of these modern fords and culverts and in the stream reaches upstream and downstream of the removed stone weir, the bottom of the channel was stabilised by building stone rapids with a gentle slope. Therefore, the longitudinal continuity of the Hołubla Stream was restored.

### Spatial and temporal structure of sampling

The research was conducted over a period of 10 years. Ten sampling sites were established in the Hołubla Stream (Fig. 1, Table A3d, A4, A5). Each study site included a 100 m stream reach. The stream was divided into two sections: UPSTREAM and DOWNSTREAM of the most downstream barrier. Sites 1–7 were located in the UPSTREAM section and sites 8–10 were located in the DOWNSTREAM section. Sampling periods were divided into two periods: BEFORE barrier removal (2010–2013), i.e., the period when the stream was divided by barriers, and AFTER (2014–2019), i.e., after barrier removal. Sampling was performed during the ‘wet season’ (spring, from the end of April to the end of May, when water flowed through the entire length of the stream) and the ‘dry season’ (summer, from mid-August to the end of September, when partial drying occurred in some years, and aquatic habitat was restricted to pools).

### Habitat descriptors

Hydromorphological parameters were measured at each site (Table A5). The substrate in each sampling site was apportioned into cobbles (256–65 mm), pebbles (64–17 mm), gravel (16–2 mm), and sand (< 2 mm). These categories were assigned according to the criteria proposed by Bain et al.^69^. Water temperature and dissolved oxygen (DO) were measured using a Multiparameter Sonde 6600 V2 (YSI Inc., Yellow Springs, OH, USA), each year during the spring and summer sampling periods (i.e., in the wet and dry season). A total of 20 replicates were collected for each site. Temperature and DO measurements were carried out from 8:00 am to 10:00 am, starting from site 1. At each site on each sampling date, to determine the maximum and average depth, water depth was measured at ten cross sections with measurements recorded every 25 cm in each section. The water flow was measured using a FlowTracker acoustic Doppler handheld flow meter (SonTek/YSI Inc., San Diego, CA USA) on each sampling date to calculate the discharge. Based on visual assessments of habitat type, three types of habitats were distinguished in the stream: (1) riffle: an area of stream characterised by shallow depths with fast, turbulent water; (2) run: an area of stream characterised by moderate currents, continuous surface, and depths greater than riffles; and (3) pools: an area of the stream characterised by deep waters and slow currents^70^. Pools in the wet season were the deepest parts of the stream, and in the dry season they were sometimes isolated. The abundance of pools in the site (approximate pools area *vs.* site area), was assessed on a four-point scale, designated by the authors, ranging from 0 to 3, where: ‘0’ indicates pools area < 5%; ‘1’ indicates pools area 5–20%, ‘2’ indicates pools area 20–35%, and ‘3’ indicates pools area > 35% of the site area.

To describe the effects of drought on the functioning of the fish population, we also used three indices: the hydrological continuity index (HCI), dryness index (DRI), and hydrological stability index (HSI) (Table A6). Table A5 presents the average values of habitat descriptors for several years (i.e., four years for the BEFORE period, and six years for the AFTER period).

### Fish sampling

Fish sampling was conducted twice a year during a 10-year period (2010–2019). At each site, 20 fish surveys were performed. Fish were sampled each year in two seasons: WET and DRY. In order to determine the regional pool of fish species, fish were caught in the mainstem river, i.e. San River, in 2012, 2017, and 2019. The fish were caught using a backpack-type electrofishing equipment (IG600T; Hans Grassl GmbH, Schönau am Königssee, Germany; 650 W DC; 1,200 W AC; 115–565 V). Each fishing crew consisted of one person operating the anode and two people catching and measuring (the total length, to the nearest 1 mm) the fish. The crew conducted single-pass electrofishing without block nets in an upstream direction. A single electrofishing pass without block nets accurately indexes the abundance of fish in small streams^71,72^, and circumvents the problems of differential capture probabilities on subsequent passes, including the potential harm to fish^71,73^. The fish were divided into the adult (AD), juvenile (JV), and young-of-the-year (YY) age classes (Table A7). The fish were identified and released at the point of capture. Relative fish abundance was calculated and recorded as the catch per unit effort (CPUE). The electrofishing CPUE was calculated and recorded as the number of fish caught per 100 m^2^ fished area (individuals (ind.) 100 m^-2^). At each site, the length of the section being fished was measured and the average width of the channel was calculated based on 10 measurements. The area of the sampling sites was calculated based on these dimensions.

### Data analysis

Statistical analyses were performed using STATISTICA v. 13.3 (TIBCO Software Inc., Palo Alto, CA, USA) and PRIMER v. 7^74^ , and CANOCO v. 5.1 (Microcomputer Power, Ithaca, NY, USA).

### Impact of barrier removal on fish communities

To illustrate changes in fish fauna over time in the context of barrier removal, the ranges (i.e., spatial distribution of stream fish) and density of fish in individual years of research were presented graphically. Then, a time series showing changes in total fish densities was constructed. Total density was presented separately for each year as the average of all catches in a given year, separately for two stream reaches (UPSTREAM: sites 1–7 and DOWNSTREAM: sites 8–10). Differences between total fish densities between the two periods (BEFORE and AFTER), for the UPSTREAM and DOWNSTREAM reaches, were analysed using the nonparametric Mann–Whitney U-test^75^.

To assess the variations in the entire fish assemblage structure, a permutational multivariate analysis of variance (PERMANOVA) (Table A8a) was performed for the entire study period (2010–2019), using a similarity matrix based on the Bray–Curtis distance of fish densities. Because of the large number of zeros in the data, analyses between samples were made using a zero-adjusted Bray–Curtis coefficient, including a virtual dummy species for all samples, prior to computing similarities^74,76^. The fish density data were log(x + 1) transformed. The PERMANOVA was run using permutation of residuals under a reduced model. Four factors were taken into account in the tested model: Reach (fixed; two levels: UPSTREAM, DOWNSTREAM), Period (fixed; two levels: BEFORE, AFTER), Season (fixed; two levels: WET, DRY), and Site (random, ten levels, nested in the Reach). Next, to assess the degree of variation in fish assemblages at the sites, pair-wise comparison procedures among all pairs of Site levels were performed separately for UPSTREAM and DOWNSTREAM reach.

### Interactions of presence/absence of barriers with seasonal flow conditions ***vs***. fish communities

In order to assess how seasonal flow conditions interact with barrier removal, changes in total fish densities were presented separately for the WET and DRY season in the time series. Differences between total fish densities between the two seasons (WET and DRY) for the UPSTREAM and DOWNSTREAM reaches were analysed using the nonparametric Mann–Whitney U-test^75^. Next, for the site samples, grouped according to the results of above tests, a similarity percentage procedure (SIMPER, based on abundance data) was performed (Table A8b). In the absence of significant differences in the U-test, the samples in the SIMPER analysis were pooled. Therefore, only three groups were distinguished, namely GrU: UPSTREAM sites, in both seasons, in the AFTER period; GrD1: DOWNSTREAM sites, in the DRY season, in both periods; and GrD2: DOWNSTREAM sites in the WET season, in both periods. This analysis breaks down the contribution of each species to the observed similarity (or dissimilarity) between the samples. This allows the identification of the species that are most important in creating the observed pattern of similarity^77,78^. To show the differences between the two periods, BEFORE and AFTER barrier removal, in relation to individual species/classes, the average density was calculated for each site, distinguishing between two hydrological seasons (WET and DRY) and presented as shade plots^74,79^ (Table A8c).

### Habitat factors determining fish communities after barrier removal

Canonical correspondence analysis (CCA)^80,81^ was applied to the species abundance data to investigate the relationship between environmental variables (i.e., habitat descriptors) and variation in fish species composition after barriers have been removed (Table A8d). Age classes of fish were also included in the analysis.

## Supplementary Information

Supplementary Information.

## Data Availability

The datasets generated during and/or analysed during the current study are available from the corresponding author on reasonable request.
