# Supplementary material for: Barrier removal and dynamics of intermittent stream habitat regulate persistence and structure of fish community
Source: Sci Rep. 2022 Jan 27;12:1512. doi: 10.1038/s41598-022-05636-7 (PMC8795198; doi:10.1038/s41598-022-05636-7)
Supplement: Supplementary file 1 — Supplementary Information. [file 41598_2022_5636_MOESM1_ESM.pdf]

**Barrier removal and dynamics of intermittent stream habitat regulate persistence and structure of fish community**

**Krzysztof Kukuła[1], Aneta Bylak[1,]***

*Corresponding author: E-mail address: abylak@ur.edu.pl (A. Bylak)

[1]Department of Ecology and Environmental Protection, University of Rzeszow, ul. Zelwerowicza 4, 35-601 Rzeszów, Poland

## Supplementary Information

**Table A1.** Pair-wise tests results from PERMANOVA of the fish assemblages  at sites in two reaches the Hołubla Stream

**Table A2.** Species-age classis contributing of the similarity in the fish communities within each of the three groups of sites (one-way SIMPER analysis).

**Table A3.** Additional information - detailed study area characteristics.

**Table A4.** Categories of precipitation and air temperature in the Hołubla Stream catchment area (the Carpathian climatic region) based on Bulletins of the Institute of Meteorology and Water Maganement.

**Table A5.** Morphological characteristics of the sampling sites.

**Table A6.** Data collection - additional explanations of the calculated indices.

**Table A7.** Age categories of the individual fish species.

**Table A8.** Data analysis - detailed description of methods.

**Table A1.** Pair-wise tests results from PERMANOVA of the fish communities at sites in two reaches (UPSTREAM, DOWNSTREAM) in the Hołubla Stream.

**a)** UPSTREAM the most downstream barrier

| **Site** | **Site** 1 | 2 | 3 | 4 | 5 | 6 | 7 |
|---|---|---|---|---|---|---|---|
| 1 | | | | | | | |
| 2 | *no test* | | | | | | |
| 3 | NS | NS | | | | | |
| 4 | *** | *** | ** | | | | |
| 5 | *** | *** | *** | NS | | | |
| 6 | *** | *** | *** | NS | NS | | |
| 7 | *** | *** | *** | NS | NS | NS | |

**b)** DOWNSTREAM the most downstream barrier

| **Site** | **Site** 8 | 9 | 10 |
|---|---|---|---|
| 8 | | | |
| 9 | NS | | |
| 10 | NS | NS | |

999 - permutations; NS – differences were non-significant;
* - P<0.05; ** - P<0.01; *** - P<0.001

**Table A2.** Species-age classis contributing of the similarity in the fish communities within each of the three groups of sites (one-way SIMPER analysis).

| **Site group (AvSim)** | **Species-age class** | **AvAbu** | **AvSim** | **AvSim/SD** | **Con (%)** | **CuCon (%)** |
|---|---|---|---|---|---|---|
| GrU: UPSTREAM sites, in both seasons, in the AFTER period (20.12%) | BB-AD | 0,73 | 8,03 | 0,66 | 39,94 | 39,94 |
| | PP-AD | 0,65 | 7,48 | 0,62 | 37,17 | 77,11 |
| | PP-JV | 0,34 | 2,19 | 0,41 | 10,91 | 88,02 |
| | BB-JV | 0,29 | 1,76 | 0,35 | 8,75 | 96,76 |
| | ST-JV | 0,10 | 0,32 | 0,18 | 1,61 | 98,37 |
| | ST-AD | 0,11 | 0,30 | 0,12 | 1,51 | 99,88 |
| | PP-YY | 0,05 | 0,02 | 0,06 | 0,09 | 99,97 |
| | BB-YY | 0,03 | 0,01 | 0,02 | 0,03 | 100,00 |
| GrD1: DOWNSTREAM sites, in the DRY season, in both periods (12.09%) | PP-AD | 0,41 | 3,43 | 0,51 | 28,36 | 28,36 |
| | BB-JV | 0,36 | 3,07 | 0,43 | 25,37 | 53,73 |
| | PP-JV | 0,44 | 2,82 | 0,41 | 23,35 | 77,08 |
| | BB-AD | 0,33 | 1,59 | 0,30 | 13,14 | 90,22 |
| | PP-YY | 0,25 | 0,61 | 0,20 | 5,01 | 95,22 |
| | BB-YY | 0,16 | 0,36 | 0,20 | 2,96 | 98,19 |
| | GG-AD | 0,08 | 0,11 | 0,13 | 0,88 | 99,07 |
| | SC-YY | 0,07 | 0,09 | 0,13 | 0,77 | 99,84 |
| | GG-YY | 0,02 | 0,02 | 0,06 | 0,16 | 100,00 |
| GrD2: DOWNSTREAM sites in the WET season, in both periods (38.03%) | BB-AD | 1,39 | 10,12 | 1,09 | 26,60 | 26,60 |
| | PP-JV | 1,38 | 8,65 | 1,24 | 22,76 | 49,36 |
| | BB-JV | 1,17 | 7,10 | 0,79 | 18,68 | 68,04 |
| | PP-AD | 1,07 | 6,30 | 0,74 | 16,56 | 84,59 |
| | PP-YY | 0,97 | 2,49 | 0,49 | 6,56 | 91,15 |
| | SC-JV | 0,53 | 0,88 | 0,34 | 2,31 | 93,47 |
| | SC-YY | 0,38 | 0,70 | 0,36 | 1,83 | 95,30 |
| | GG-AD | 0,32 | 0,51 | 0,28 | 1,33 | 96,63 |
| | BB-YY | 0,47 | 0,43 | 0,22 | 1,13 | 97,76 |
| | GG-JV | 0,32 | 0,34 | 0,25 | 0,88 | 98,64 |
| | ST-AD | 0,04 | 0,15 | 0,07 | 0,40 | 99,04 |
| | BA-YY | 0,13 | 0,11 | 0,18 | 0,29 | 99,33 |
| | LL-YY | 0,10 | 0,11 | 0,18 | 0,28 | 99,60 |
| | ST-JV | 0,06 | 0,09 | 0,12 | 0,24 | 99,85 |
| | ST-YY | 0,06 | 0,04 | 0,15 | 0,10 | 99,95 |
| | SC-AD | 0,05 | 0,01 | 0,05 | 0,03 | 99,97 |
| | LL-JV | 0,07 | 0,01 | 0,08 | 0,03 | 100,00 |

Each species contribution (Con) to the total similarity within the site is given as a percentage, and the cumulative contribution (CuCon) of each taxon. Column 5 gives the ratio of average similarity (AvSim) divided by standard deviation (SD); Average abundance, log(x+1) transformed (AvAbu);

Species abbreviations: AB, *Alburnoides bipunctatus*; BA, *Barbus barbus*; BB, *Barbatula barbatula*; GG, *Gobio gobio;* LL, *Leuciscus leuciscus*; PF, *Perca fluviatilis;* PP, *Phoxinus phoxinus*; RA, *Rhodeus amarus*; SC, *Squalius cephalus;* ST, *Salmo trutta fario*;

Age class abbreviations: YY, young-of-the-year (fry during their first year of life); JV, juveniles; AD, adults; sites: 1–10; the Periods: B - BEFORE and A - AFTER barrier removal.

**Table A3.** Additional information - detailed study area characteristics.

| **Detailed study area characteristics** |
|---|
| **a)**<br>• The section of the San River downstream the mouth of the Hołubla Stream is largely regulated.<br>• Modifications of the San River channel were implemented from the second half of the 19th century, and these modifications covered, among others, the ~70-km reach of the river, located approximately 15 km downstream of the mouth of the Hołubla Stream.<br>• In this section, the meanders along the river were removed to make the river straighter; thus, the San River has been shortened by almost 40 km. Consequently, there has been more than a two-fold increase in the slope of the riverbed; the San River channel cut into alluvial deposits and the riverbed bottom was lowered by almost 4 m (Dynowska and Maciejewski, 1991).<br>• Owing to the drainage effect of the San River and the lowering of the groundwater level, most of the small tributaries of the San River, including the Hołubla Stream, in their downstream sections near the mouth to the mainstem river, disappear under dry conditions (Fig. 2a). |
| **b)**<br>• The Hołubla Stream catchment area (Fig. 1) belongs to the Carpathian climatic region, with an average annual temperature for 1971–2000 of 8°C, and an average annual rainfall for 1971–2000 of 600–800 mm. Using the rainfall and thermal scales (IMWM, 2020), average rainfall and temperatures were assessed for the summer (June–August) and spring (March–May) months.<br>• In this part of the Carpathians, individual summers in the research period 2010–2019 were defined as very dry, dry or normal (supplementary Table A4).<br>• The summer months were warm, very warm, or extremely warm (Table A3; IMWM, 2020).<br>• Individual springs in the research period 2010–2019 were defined as dry, normal, or wet (Table A3). Consequently, the FLOW (spring) and DRY (summer) states were distinguished in each year. |
| **c)**<br>• The catchment of the Hołubla Stream has a high natural value (part of the Natura 2000 network, and nature reserve). Almost the entire Hołubla Stream basin is forested (99% of the basin). The dominant plant communities are the lime–oak–hornbeam forest (*Tilio-carpinetum*) and the Carpathian beech forest (*Dentario glandulosae-Fagetum*).<br>• The Hołubla Stream is 5.89-km long, and its catchment area is 8.68 $km^2$. The flow in the non-drying parts of the middle stream ranges from approximately 5 L $s^{-1}$ in summer to 150 L $s^{-1}$ in spring. The water in the Hołubla Stream is of very good quality (well-oxygenated water, i.e., 7.0–10.3 $mgO_2$ $L^{-1}$; water conductivity, 290–495 µS $cm^{-1}$), with negligible anthropogenic pollutants. |
| **d)**<br>• In the upper course of the Hołubla Stream (sites 1 and 2), the streambed substrate was stony gravel, predominantly cobbles and pebbles. The channel width varied from 1 to 2 m. During the 2010–2019 study period, the sites usually presented a perennial flow of water; however, in the extremely warm and dry years, the upper course of the stream dried (Table A4, A5).<br>• In the middle course of the Hołubla Stream (sites 3-7), the bottom substrate mostly comprised pebbles and cobbles (sites 3-6), or cobbles, gravel, and sand (site 7). Under dry conditions, water formed a chain of isolated pools. At sites 3 and 4, pools were up to 60 cm in depth, and at sites 5 and 6, there were several more extensive pools (4–8 m in length) with deeper water (up to 80 cm).<br>• In the downstream course of the Hołubla Stream (sites 8-10), stones dominated the bottom substrate.<br>• Sites 9 and 10 were located within the San River alluvia (Fig. 1).<br>• Under dry conditions, stream at site 8 and 9 most often dried up, or isolated pools remained, and at site 10, the stream reach was usually completely dry for several weeks (Table A5). |

**Table A4.** Categories of precipitation and air temperature in the Hołubla Stream catchment area (the Carpathian climatic region) based on Bulletins of the Institute of Meteorology and Water Maganement (IMWM, 2020) data.

| Year | Season | Precipitation | Temperature |
|---|---|---|---|
| **2010** | spring | **normal** | **normal** |
| | summer | **normal** | **warm** |
| **2011** | spring | **dry** | **warm** |
| | summer | **normal** | **warm** |
| **2012** | spring | **dry** | **warm** |
| | summer | **dry** | **very warm** |
| **2013** | spring | **wet** | **normal** |
| | summer | **dry** | **warm** |
| **2014** | spring | **wet** | **warm** |
| | summer | **normal** | **warm** |
| **2015** | spring | **wet** | **normal** |
| | summer | **very dry** | **extremely warm** |
| **2016** | spring | **normal** | **warm** |
| | summer | **normal** | **very warm** |
| **2017** | spring | **normal** | **warm** |
| | summer | **very dry** | **very warm** |
| **2018** | spring | **dry** | **very warm** |
| | summer | **very dry** | **very warm** |
| **2019** | spring | **normal** | **warm** |
| | summer | **very dry** | **very warm** |

**The rainfall scale:**

- **very dry** (50-75% of the AAR)
- **dry** (< 75-90% of the AAR)
- **normal** (90%–110% of the AAR)
- **wet** (>110% of the AAR)

AAR - an average annual rainfall for 1971–2000: 600–800 mm

**The thermal scale:**

- **normal** (AAT - 0.5 *SD* < T ≤ AAT + 0.5 *SD*)
- **warm** (AAT + 1.0 *SD* < T ≤ AAT + 1.5 *SD*)
- **very warm** (AAT + 1.5 *SD* < T ≤ AAT + 2.0 *SD*)
- **extremely warm** (T > AAT + 2.5 *SD*)

AAT- an average annual temperature for 1971–2000: 8°C
T – an average temperature in a given year
*SD* – standard deviation

**Table A5.** Morphological characteristics of the sampling sites.

| | | | Mean | | | | | | | | | | | | |
|---|---|---|---|---|---|---|---|---|---|---|---|---|---|---|---|
| **Site** | **Season** | **DMT (m)** | **WID (m)** | **Bottom substrate (%) C/P/G/S** | **SHC (%)** | **BDN** | **LWD*** | **DRI B/A** | **Depth (cm) B/A** | **Discharge (L $s^{-1}$) B/A** | **HCI B/A** | **HSI B/A** | **Pools* B/A** | **Temperat ure (°C) B/A** | **Dissolved Oxygen (mg $L^{-1}$) B/A** |
| **1** | WET | 3400 | 2.0 | 45/45/5/5 | 90 | 1/0 | 1 | 1/1 | 15/11 | 90/57 | 2/2 | 0/0 | 1/1 | 13.5/13.4 | 10.3/9.9 |
| | DRY | | | | | | | 0.75/0.83 | 10/4 | 11/3 | 1.0/0.67 | 2/0 | 3/0.8 | 13.9/14.9 | 8.9/8.7 |
| **2** | WET | 3015 | 2.0 | 45/40/10/5 | 50 | 1/0 | 1 | 1/1 | 15/11 | 90/57 | 2/2 | 0/0 | 1/1 | 13.7/13.4 | 10.3/9.9 |
| | DRY | | | | | | | 1/1 | 10/4 | 11/3 | 1.0/0. 67 | 2/0 | 3/0.8 | 14.0/15.1 | 8.9/8.7 |
| **3** | WET | 2675 | 2.5 | 40/40/15/5 | 90 | 1/0 | 3 | 1/1 | 20/13 | 103/62 | 2/2 | 0/0 | 3/3 | 13.7/13.6 | 10.3/9.8 |
| | DRY | | | | | | | 1/1 | 10/10 | 11/3 | 1.25/0.67 | 2/0 | 3/3 | 14.0/15.2 | 9.1/8.7 |
| **4** | WET | 2035 | 2.5 | 40/40/15/5 | 90 | 1/0 | 3 | 1/1 | 20/14 | 103/73 | 2/2 | 0/0 | 3/3 | 13.8/13.6 | 10.2/9.9 |
| | DRY | | | | | | | 1/1 | 10/10 | 11/5 | 1.25/0.83 | 2/0 | 3/3 | 14.1/15.2 | 9.0/8.9 |
| **5** | WET | 1715 | 3.0 | 20/50/15/15 | 40 | 1/0 | 3 | 1/1 | 25/18 | 102/72 | 2/2 | 0/0 | 3/3 | 14.0/13.7 | 10.2/9.9 |
| | DRY | | | | | | | 1/1 | 10/12 | 11/5 | 1.25/0.83 | 2/0 | 3/3 | 14.2/15.6 | 8.9/8.9 |
| **6** | WET | 1335 | 3.0 | 25/50/15/10 | 80 | 1/0 | 3 | 1/1 | 24/19 | 102/72 | 2/2 | 0/0 | 3/3 | 13.9/13.8 | 10.2/9.9 |
| | DRY | | | | | | | 1/1 | 10/11 | 11/4 | 1.25/0.83 | 2/0 | 3/3 | 14.2/15.9 | 8.9/8.8 |
| **7** | WET | 1145 | 3.5 | 15/40/30/15 | 50 | 1/0 | 2 | 1/1 | 23/18 | 102/71 | 2/2 | 0/0 | 2/2 | 14.1/13.9 | 10.1/9.8 |
| | DRY | | | | | | | 1/0.83 | 7/8 | 8/2 | 0.75/0.5 | 2.2/0 | 2/2 | 14.6/16.0 | 8.6/8.6 |
| **8** | WET | 985 | 3.0 | 50/35/10/5 | 60 | 0/0 | 1 | 1/1 | 28/21 | 111/72 | 2/2 | 0/0 | 1/1 | 14.3/14.3 | 10.0/9.8 |
| | DRY | | | | | | | 0.75/0.83 | 7/4 | 6/0.3 | 0.75/0.17 | 2.2/1.3 | 0.8/0 | 14.8/16.6 | 7.9/8.0 |
| **9** | WET | 690 | 3.5 | 25/50/10/5 | 30 | 0/0 | 1 | 1/1 | 29/20 | 119/70 | 2/1.8 | 0/0 | 1/1 | 14.7/14.8 | 10.1/9.7 |
| | DRY | | | | | | | 0.5/0.5 | 4/2 | 4/0.3 | 0.75/0.17 | 7.2/6.2 | 0.5/0 | 14.9/18.3 | 8.0/7.2 |
| **10** | WET | 290 | 3.5 | 5/60/25/10 | 80 | 0/0 | 2 | 1/1 | 28/20 | 119/70 | 2.0/2.0 | 0.5/0 | 2/2 | 14.9/14.9 | 9.8/9.8 |
| | DRY | | | | | | | 0.5/0.17 | 3/1 | 3/0 | 0.25/0 | 8.7/7.7 | 0.5/0 | 16.0/19.0 | 8.0/7.0 |

Periods: B- BEFORE barrier removal (2010-2013), A- AFTER barrier removal (2014-2019); C – cobbles, P – pebbles, G – gravel, S – sand; DMT – distance from the mouth, WID – channel width, SHC - shade cover , BDN – barrier downstream the site, where ‘0’ indicates absence, ‘1’ indicates presence; LWD - large woody debris, DRI - dryness index, HCI - hydrological continuity index, HSI - hydrological stability index; *- four-point scale (range 0–3), where ‘0’ indicates lowest level; **- four-point scale (range 0–3), approximate pools area *vs*. site area, in %: ‘0’ indicates <5%, ‘1’ indicates 5-20%, ‘2’ indicates 20-35%, ‘3’ indicates pools area >35% of the site area.

**Table A6.** Data collection - additional explanations of the calculated indices.

| Abbre-viation | Full name | Characteristics |
|---|---|---|
| HCI | Hydrological continuity index | • indirectly characterises the possibility of fish movement at the stream reach at a given site.<br>• range 0-2<br>• was assessed when the fish were sampled.<br>• in the absence of water or the presence of isolated pools (separated by dry riffles and runs) at the site, the HCI value was '0'.<br>• for a strip of water with a depth not exceeding 2 cm in the riffles and runs, the HCI value was '1'.<br>• when there was more flowing water, the HCI value was ‘2’. |
| DRI | Dryness index | • Data were collected from each site at the time of sampling<br>• the value ‘1’ was assigned for the presence of water at the stream reach<br>• for a completely dry stream bed, the assigned value was '0'. |
| HSI | Hydrological stability index | • To estimate the HSI, two half years were considered.<br>• The flow conditions (the higher flow period) were December–May<br>• The dry conditions (the low flow period) were June–November.<br>• HSI defined the duration in a given year of the study (counted in weeks for each sampling site) when water was absent in the stream reach. |

**Table A7.** Age categories of the individual fish species (Brylińska 2000,  Kukuła K. and Bylak A., unpubl. data ).

| **Fishes** | **Age categories** | | |
|---|---|---|---|
| | **AD** | **JV** | **YY** |
| Siberian sculpin *Cottus poecilopus* | >80 | 80-51 | ≤50 |
| Brown trout *Salmo trutta fario* | >170 | 170-101 | ≤100 |
| Common minnow *Phoxinus phoxinus* | >70 | 70-51 | ≤50 |
| Stone loach *Barbatula barbatula* | >80 | 80-51 | ≤50 |
| Chub *Squalius cephalus* | >180 | 180-81 | ≤80 |
| Barbel *Barbus barbus* | >180 | 180-81 | ≤80 |
| Gudgeon *Gobio gobio* | >70 | 70-51 | ≤50 |
| Dace *Leuciscus leuciscus* | >180 | 180-81 | ≤80 |
| Bitterling *Rhodeus amarus* | >60 | 60-51 | ≤50 |
| Spirlin *Alburnoides bipunctatus* | >80 | 80-61 | ≤60 |
| Perch *Perca fluviatilis* | >150 | 150-101 | ≤100 |

The criteria for the allocation were based on the total length (TL, mm).
YY: fry during their first year of life, JV: fish that have not reached sexual maturity, AD: adult (mature) fish that have reached sexual maturity.

**Table A8.** Data analysis - detailed description of methods.

| Method | Description |
|---|---|
| **a)** PERMA NOVA | • A highly appropriate measure for most ecological data (being counts or other measures of abundance of species), as they often tend to be overdispersed, with heavily right-skewed distributions and a plethora of zeros. In addition, the number of variables (usually species or taxa) often far exceeds the number of sampling units, making traditional statistical approaches either problematic or impossible.<br>• The methods in PERMANOVA allow multivariate data to be modelled, analysed, and tested based on any resemblance measure of choice, and all tests of hypotheses are performed using permutation techniques (Anderson et al. 2008). |
| **b)** SIMPER | • Was run to identify fish species/classes that are most likely to account for the similarities within groups, i.e., GrU: UPSTREAM sites, in both seasons, in the AFTER period; GrD1: DOWNSTREAM sites, in the DRY season, in both periods; and GrD2: DOWNSTREAM sites in the WET season, in both periods.<br>• The percentages of each fish species/class in the Bray–Curtis similarity index within the group of sites a were determined. These values indicated the species that were characteristic to each site group. The typical species in a site group were identified using samples acquired from a site with constant abundance and a high ratio of similarity contribution (AvSim) to SD (AvSim/SD) for that site (Clarke and Gorley, 2015). |
| **c)** Shade plots | • Shade plots prepared for the 10 Sites × Period combinations (x-axis) and 21 species/classes (y-axis) are shown, separately for WET and DRY seasons.<br>• Shade plots have the capacity not only to identify common patterns in groups of taxa which appear to be determining sample group structures (as an adjunct to categorical, similarity-based tools such as SIMPER (Clarke, 1993)), but also to interpret continuous multivariate assemblage changes at the level of individual species (Clarke et al., 2014). |
| **d)** CCA | • The analysis was performed on the AFTER barrier removal data, as this factor would hinder the correct assessment of the impact of other environmental factors. The analyses included samples from sites where fish appeared at least once. Young cyprinid fish (CYP) occurring in the lower section of the Hołubla Stream, belonging to the species associated with the mainstem rivers, for the CCA analysis were summed up into one category (i.e., CYP-JV+YY).<br>• Eighteen environmental variables were initially considered for investigation; however, their number was reduced to avoid multicollinearity. Pearson's correlation coefficients were calculated for all pairs of environmental variables to identify the sources of redundancy.<br>• Ultimately, six variables (i.e., distance from the mouth, pebbles, water temperature, pools, discharge, and HCI) were used. Species/classes that appeared in less than 5% of the samples were removed from the set (Arrington and Winemiller, 2003).<br>• The collected data was first analysed through gradient length calculations using detrended canonical correspondence analysis (DCCA). The length of the longest gradient in DCCA was 3.7. Consequently, CCA was applied to assess the dependence of fish abundance on environmental variables (ter Braak and Šmilauer, 2012).<br>• The data were centred and standardised. The percentage of environmental data describing the fraction of substrate were arcsine transformed to ensure that the assumptions of the model were met.<br>• The CCA was conducted using a forward-selection procedure. The significance of the CCA axes in explaining species diversity was determined, as was the significance of the effects of specific variables on model ordering, based on a Monte Carlo test with 999 random permutations.<br>• The importance of environmental variables for fish species is described by the length of the arrows in the CCA biplot. The length of the environmental vector indicates the strength of the correlation, and its direction indicates its relationship with the species.<br>• Fish species plotted in the same direction from the origin as an environmental vector are positively correlated with that variable, and a species plotted in the opposite direction indicates a negative relationship (ter Braak and Šmilauer, 2012). |

**References**


1. Anderson, M. J., Gorley, R. N. & Clarke, K. R. PE*RMANOVA+ for PRIMER: Guide to Software and Statistical Methods*. PRIMER-E, Plymouth. (2008).
2. Arrington, D. A. & Winemiller, K. O. Diel changeover in sandbank fish assemblages in a neotropical floodplain river. *J. Fish Biol.* **63**, 442–459. (2003).
3. Brylińska, M. *Freshwater Fishes of Poland*. PWN, Warszawa. (2000). [in Polish]
4. Clarke, K. R. Non-parametric multivariate analyses of changes in community structure. *Aust. J. Ecol.* **18**, 117–143. (1993).
5. Clarke, K. R. & Gorley, R. N. *PRIMER v.7: User Manual/Tutorial*. PRIMER-E, Plymouth. (2015).
6. Clarke, R. K., Tweedley, J. R. & Valesini, F. J. Simple shade plots aid better long-term choices of data pre-treatment in multivariate assemblage studies. J. Mar. Biolog. Assoc. U.K. 94, 1 – 16. (2014).
7. Dynowska, I. & Maciejewski, M. (Eds.). Upper Vistula Basin. PWN, Warszawa-Kraków. (1991).
8. IMWM. Bulletins of the Institute of Meteorology and Water Maganement. National Research Institute. https://www.imgw.pl/en (2020).
9. ter Braak, C.J.F. & Šmilauer, P. *Canoco Reference Manual and Users Guide: Software for Ordination (Version 5.0).* Ithaca, NY: Microcomputer Power. (2012).
